# Reconstruction of the lncRNA-miRNA-mRNA network based on competitive endogenous RNA reveals functional miRNAs and lncRNAs in burns and keloids

**DOI:** 10.1371/journal.pone.0320855

**Published:** 2025-04-09

**Authors:** Yueru Wang, Zhichao Wang, Jiaojiao Pan, He Wang, Ziwen Lei, Jing Liu, Junbo Zou, Haizhen Lv, Fei Luan

**Affiliations:** 1 College of Medicine and Pharmacy, Shaanxi Institute of International Trade & Commerce, Xi’an, Shaanxi, P.R. China; 2 Shaanxi Province Key Laboratory of New Drugs and Chinese Medicine Foundation Research, School of Pharmacy, Shaanxi University of Chinese Medicine, Xi’an, Shaanxi, P.R. China; 3 Department of Pharmacy, Shaanxi Provincial Hospital of Tuberculosis Prevention and Treatment, Xi’an, P.R. China; University of Pennsylvania, United States of America

## Abstract

**Backgrounds:**

Long non-coding RNAs (lncRNAs) exert their pharmacological functions by serving as sponges for related microRNAs (miRNAs), thereby modulating gene expression. Nevertheless, the regulatory roles of the lncRNA-mediated competing endogenous RNA (ceRNA) mechanism in the interplay between burns and keloids remain largely elusive.

**Objective:**

To construct the ceRNA regulatory network of burns, leveraging network pharmacology and bioinformatics analyses.

**Results:**

3576 DELs (Differentially Expressed lncRNAs), 1427 DEMis (Differentially Expressed miRNAs), and 2555 DEMs (Differentially Expressed mRNAs) were identified as differentially expressed genes. A ceRNA network composed of DELs-DEMis-DEMs in burns and keloids was constructed, with a prominent sub-network consisting of 23 DELs, 330 DEMs, and 8 DEMis. Subsequently, the clusterProfiler package in the R programming language was utilized to perform Gene Ontology (GO) and Kyoto Encyclopedia of Genes and Genomes (KEGG) pathway analyses. The sub-network within the ceRNA network was extracted, in which three lncRNAs, namely lnc-WRB, lnc-SCNN1G, and LINC00271, and three miRNAs, namely hsa-miR-21, hsa-miR-34a, and hsa-miR-155, were identified as key genes.

**Conclusion:**

All nodes within the sub-ceRNA network exert either a direct or an indirect influence on the pathological processes of burns and post-burn keloids. The current study successfully constructed the ceRNA network in burns and keloids and provided a potentially novel perspective on the DELs-DEMis-DEMs ceRNA network, contributing to the elucidation of the ceRNA regulatory mechanisms in the pathogenesis of burns and keloids. Nevertheless, systematic and rigorous experimental validations are indispensable to confirm our findings.

## 1. Introduction

It is reported that the skin is the largest human organ, holding preeminent importance for human health and welfare [1]. Burns are defined as skin injuries induced by exposure to thermal sources, chemical substances, electrical currents, radiation, and other factors [2]. According to the World Health Organization (WHO), an estimated 180,000 deaths every year occur due to burn injuries. The research on the global burden of disease revealed that approximately nine million new cases of burn injury required medical attention in 2019 [3]. Contact with flames, hot or boiling liquids constitutes over 88% of these thermal burns [2]. Keloids and Hypertrophic scars (HS) are prevalent complications of severe burns, characterized by abnormal fibroproliferative growth during the wound-healing process [4], which create a problem of enormous magnitude. The U.S. alone expends at least $4 billion annually on the treatment of these cases [5]. Severe burns invariably trigger systemic inflammatory and stress responses, inflicting acute insults on the body and subsequently leading to the disruption of bone and skeletal muscle [6], acute kidney injury [7, 8] and protein-losing enteropathy [9]. Patients, particularly children with severe burns, are frequently subjected to intense psychological stress, and their psychological needs deserve serious consideration from society [10].

Gene therapy has attracted a lot of attention for treating burns, keloids and HS. Skin grafting results in an ideal clinical effect that has been reported frequently [11]. In recent years, profound insights have been gained into the role of skin stem cells in maintaining skin homeostasis and facilitating reparative wound healing. Moreover, researchers have explored a diverse range of techniques for the repair of severe burns and chronic wounds, such as various types of skin grafts, scaffolds, and biologics. The development of next-generation skin substitutes, gene-editing techniques, and the utilization of bioprinting and advanced biomaterials, in combination with autologous cell technology, holds great potential for more effectively reconstructing the structure and function of human skin [1]. However, it is still ambiguous about the recovery mechanism of denatured dermal function. Obtaining effective therapeutics represents a pivotal step in elucidating the molecular nature of burns and keloids.

Increasing attention has been drawn to the regulatory network consisting of lncRNAs, miRNAs, and mRNAs, which are implicated in the pathological processes of burns and keloids [12]. lncRNA, with a length exceeding 200 nucleotides, is defined as a non-coding RNA transcript present in the nucleus and cytoplasm of eukaryotic cells. Clinical and experimental studies have reported that several lncRNAs, such as CCAT2 [13], AK077216 [14], COL1A2-AS1 [15], and H19 [16], could play an important role in the regulation of burns and keloids. Additionally, miRNA, a non-coding RNA, suppresses the expression of target genes by competitively binding to the response elements of mRNA. This regulatory network of miRNAs and mRNAs has influenced a diverse array of biological and pathological processes [17]. Liang et al. analyzed the expression of miRNAs in patients with denatured dermis, compared with normal skin, 66 miRNAs, 34 down-regulated while 32 up-regulated all of which were found to be differentially regulated and associated with via several signaling pathways [11]. Furthermore, the ceRNA hypothesis, put forward by Salmena and colleagues in 2011, has been demonstrated to be implicated in the development of burns and keloids [18]. The COL1A2-AS1/miR-21/Smad pathway inhibits hypertrophic scar formation in Nong’s report serves as the target of treatment for HS [15]. As reported by Quan et al. [19], during osteoclastogenesis, lncRNA-AK131850 acts as a sponge for miR-93-5p, significantly augments the secretion of VEGFα, and facilitates vasculogenesis in wound healing.

Nevertheless, to date, no relevant research on the ceRNA mechanism in the crosstalk between burns and keloids based on network pharmacology has been retrieved. In this paper, gene expression data were retrieved from NCBI GEO datasets, and the expression profiles of lncRNAs, miRNAs, and mRNAs between the healthy normal skin group and patients with burns or keloids were analyzed. A total of 23 lncRNAs, 8 miRNAs, and 330 mRNAs were filtered to establish the ceRNA network consisting of lncRNA-miRNA-mRNA. From this network, a sub-network was constructed, which indicated that hsa-miR-21, hsa-miR-34a, and hsa-miR-155 are crucial microRNAs implicated in burns and keloids. lnc-WRB was involved in regulating hsa-miR-21 and hsa-miR-155 sub-networks, while lnc-SCNN1G, and LINC00271 were involved in both hsa-miR-21 and hsa-miR-34a sub-networks.

## 2. Materials and methods

### 2.1 Collection of raw data

The mRNA expression data of Homo sapiens, which consisted of 104 severe burn patients and 13 healthy control patients, were downloaded from the NCBI GEO database (accession number: GSE77791, https://www.ncbi.nlm.nih.gov/geo/query/acc.cgi?acc=GSE77791) based on the GPL570 platform. miRNA expression data containing 6 healthy control patients and 8 patients with keloid were collected from NCBI GEO (GSE113620, https://www.ncbi.nlm.nih.gov/geo/query/acc.cgi?acc=GSE113620) from platform GPL19117, and lncRNA microarray data for Homo sapiens containing 3 normal skin tissues and 3 patients with keloids from NCBI GEO (GSE83286, https://www.ncbi.nlm.nih.gov/geo/query/acc.cgi?acc=GSE83286) from platform GPL19612. As the data were sourced from the GEO database, no additional approval from the Ethics Committee was required.

### 2.2 Screening of differentially expressed lncRNAs, miRNAs and mRNAs

The differentially expressed lncRNAs (DELs), miRNAs (DEMis), and mRNAs (DEMs) between the healthy control group and the burns/keloid groups were identified using the limma package [20] in R programming language 4.3.1 (https://www.r-project.org) under the principle of t-test. The DELs, DEMis and DEMs were filtered according to the *P*-values <  0.05 and fold change (log_2_FC) > Mean (log_2_FC) +  2 * SD (log_2_FC). In order to visualize the DELs, DEMis and DEMs, volcano maps and heat maps were generated by the employment of the ggplot2 [21] and pheatmap [22] packages in the R programming language.

### 2.3 Prediction of target lncRNAs and mRNAs of DEMis

Firstly, the lncRNAs were annotated in UCSC Genome Browser (http://genome.ucsc.edu/), which introduced a novel method for visualizing the interactions between genomic regions [23].The LncBase Predicted v.2 of DIANA Tools was utilized to identify the interactions between lncRNAs and miRNAs [24] and these interactions were further validated by the RNAhybrid program [25]. The predicted lncRNAs were further filtered by matching them with the previously selected DELs, and subsequently, the information on DELs-DEMis pairs was obtained. Next, the targeted mRNA of DEMis were retrieved from three widely used databases including MiRBase [26], MirTarBase [27], and Targetscan [28]. The predicted mRNAs were further filtered by matching them with the previously selected DEMs, and thereafter, the interaction information of DEMis-DEMs was obtained. Finally, the pairs of DELs-DEMis and DEMis-DEMs were constructed.

### 2.4 Reconstruction of DELs-DEMis-DEMs network

The Cytoscape 3.10.2 software (https://cytoscape.org/) was utilized to visualize and reconstruct the DELs-DEMis-DEMs network by integrating all the co-expression competing triplets identified previously.The degrees of all nodes in the network were calculated, based on which the sub-networks were further constructed.

### 2.5 Functional enrichment analysis

With the help of the clusterProfiler package [29] in the R programming language, functional analysis, encompassing the Gene Ontology (GO) Biological Processes, the Kyoto Encyclopedia of Genes and Genomes (KEGG) pathway, was performed. This analysis aimed to provide a clearer understanding of the underlying biological and pathological processes of DEMs within the DELs-DEMis-DEMs network.

## 3. Results

### 3.1 Screening results of DELs in Keloids

**Fig 1:**
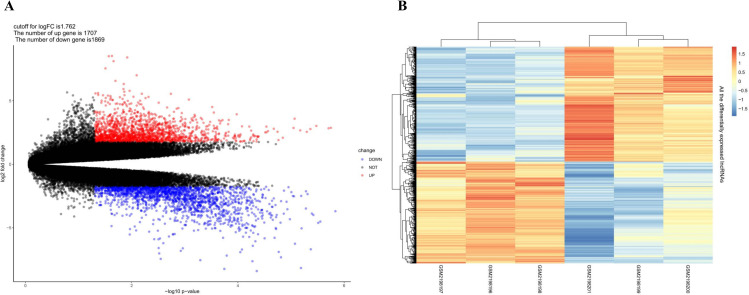
Volcano Plot and Heatmap of DELs. A. Volcano map of DELs. Red dots represent up-regulated genes, and blue dots represent down-regulated genes; B. Heatmap of DELs. The left-most three samples were derived from the normal skin tissue group, and the right-most three samples were from the keloid group. Blue color indicates low expression, while red color indicates high expression.

The expression levels of lncRNAs in three normal skin tissues and three patients with keloids were analyzed. Based on the aforementioned screening criteria, the cutoff value for the log2-fold change (log2FC) of lncRNAs was set at 1.762. A total of 1707 (47.73%) up-regulated and 1869 (52.27%) down-regulated lncRNAs were identified. In Table 1, we provided the top 25 up-regulated and 25 down-regulated ones including their names, log2FC value, P-value, and FDR values. The complete list of DELs was also presented in S1 Table. All expression levels of lncRNAs were standardized to the average log2-expression level. In Fig 1A, a volcano map illustrating the traits of all the DELs on the correlation of –log10 (p-value) and log2FC was provided, together with a heat map of DELs as shown in Fig 1B from which the difference between normal skin and keloid groups was visually displayed. As show in Table 1 and Fig 1, the log2FC value of down-regulated lncRNAs are significantly larger than those of up-regulated lncRNAs, suggesting that the former play a more crucial role in regulating the burn-related process.

**Table 1:** The top 50 lncRNAs in keloids samples.

Top 25 up-regulated lncRNAs				Top 25 down-regulated lncRNAs			
Name	Log_2_FC	*P *Value	FDR	Name	Log_2_FC	*P* Value	FDR
A_23_P372988	2.8697	1.78E-06	4.13E-02	CUST_26181_PI429545380	-3.6976	1.46E-06	4.13E-02
CUST_32954_PI429545384	2.8352	1.96E-06	4.13E-02	A_33_P3280845	-4.5129	2.81E-06	4.13E-02
A_33_P3299122	3.0214	3.34E-06	4.13E-02	CUST_18604_PI429545402	-4.611	3.07E-06	4.13E-02
CUST_45784_PI429545384	2.7763	4.31E-06	4.13E-02	GRM6	-3.9872	3.79E-06	4.13E-02
EPCAM	2.5551	5.05E-06	4.13E-02	TMEM200A	-3.8803	4.05E-06	4.13E-02
CUST_35509_PI429545395	2.6714	6.69E-06	4.13E-02	SFRP4	-6.6943	4.24E-06	4.13E-02
DEPDC4	3.5880	8.56E-06	4.30E-02	CUST_81669_PI429545388	-2.3467	4.88E-06	4.13E-02
RRP1B	2.8607	8.96E-06	4.30E-02	FBLN7	-2.1274	5.22E-06	4.13E-02
OBSCN	2.0886	1.43E-05	4.30E-02	CUST_3520_PI429545410	-3.4831	5.24E-06	4.13E-02
LNX1	1.9098	1.72E-05	4.30E-02	XR_241827.1	-5.0459	6.31E-06	4.13E-02
PARD6B	2.5599	1.79E-05	4.30E-02	CCDC195	-2.4903	6.43E-06	4.13E-02
CUST_43690_PI429545384	2.7986	2.11E-05	4.30E-02	ASPN	-8.1672	6.49E-06	4.13E-02
FR060718	2.6135	2.71E-05	4.30E-02	SFRP4	-6.255	8.42E-06	4.30E-02
SCNN1G	2.5200	2.90E-05	4.30E-02	MYO1B	-2.5209	8.77E-06	4.30E-02
PARP11	2.9672	2.90E-05	4.30E-02	PLXDC1	-2.4992	9.21E-06	4.30E-02
CHRM3	2.5099	3.05E-05	4.30E-02	CUST_47924_PI429545406	-2.1945	9.84E-06	4.30E-02
RHCG	2.1931	4.05E-05	4.30E-02	C5orf13	-4.3245	1.05E-05	4.30E-02
psiTPTE22	2.1461	4.21E-05	4.30E-02	LOC100128035	-1.8038	1.11E-05	4.30E-02
FR060718	2.2034	4.26E-05	4.30E-02	CUST_18765_PI429545388	-3.5428	1.17E-05	4.30E-02
CUST_79271_PI429545399	2.1797	4.26E-05	4.30E-02	CUST_19827_PI429545388	-2.9501	1.21E-05	4.30E-02
ZMIZ1	1.9625	4.44E-05	4.30E-02	CUST_12988_PI429545388	-2.0748	1.36E-05	4.30E-02
WRB	1.7921	4.45E-05	4.30E-02	BMPER	-3.891	1.37E-05	4.30E-02
TSKS	2.2348	4.69E-05	4.30E-02	CUST_66562_PI429545376	-2.9038	1.54E-05	4.30E-02
LINC00271	2.2072	4.93E-05	4.30E-02	ASPN	-7.325	1.54E-05	4.30E-02
CUST_57658_PI429545402	1.8182	5.08E-05	4.30E-02	SCG2	-4.5965	1.57E-05	4.30E-02

### 3.2 The DEMis screening results in Keloids

**Fig 2:**
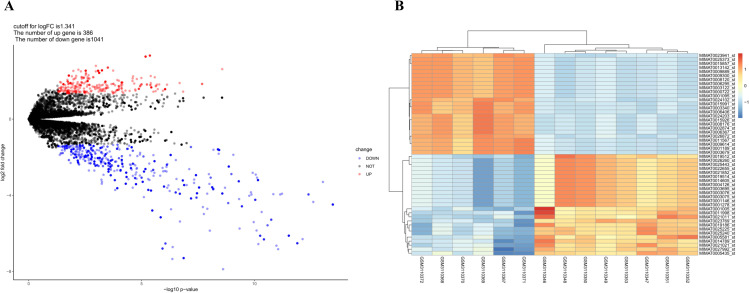
Volcano Plot and Heatmap of DEMis. A. Volcano Plot of DEMis. Red dots represent up-regulated genes, and blue dots represent down-regulated genes; B. Heatmap of DEMis. The left-most six samples were obtained from the healthy control patients’ group, and the right-most eight samples were from the keloid group. Blue color denotes low expression, while red color denotes high expression.

Based on the screening criteria, the cutoff value for log_2_FC of miRNAs was set at 1.341. A total of 386 (27.05%) up-regulated miRNAs and 1041 (72.95%) down-regulated miRNAs were identified. We outlined the top 25 up-regulated and top 25 down-regulated DEMis in Table 2. The complete list of differentially expressed miRNAs (DEMis) was also presented in S2 Table. In Fig 2A, the distribution of miRNAs on the correlation of –log_10_ (p-value) and log_2_FC was displayed by a volcano map. We also generated the heat map of DEMis, as shown in Fig 2B, presenting the difference between healthy control and keloid groups directly. The expression levels of all miRNAs were normalized to the average log2-expression level. The difference of down-regulated miRNAs seems more significant than the up-regulated ones while the former is associated with a bigger log2FC value and a smaller FDR value, see Table 2 and Fig 2.

**Table 2:** The top 50 miRNAs in keloids samples.

25 up-regulated miRNAs				25 down-regulated miRNAs			
Name	Log_2_FC	*P *Value	FDR	Name	Log_2_FC	*P *Value	FDR
hsa-miR-5001-5p	1.8069	7.40E-07	8.25E-05	hsa-miR-370-3p	-4.4863	3.36E-14	1.22E-10
hsa-miR-638	1.4385	2.99E-06	2.50E-04	hsa-miR-542-5p	-3.9561	4.01E-13	9.72E-10
hsa-miR-182-5p	1.6651	3.69E-06	2.96E-04	hsa-miR-503-5p	-6.5202	1.25E-12	2.17E-09
hsa-miR-4695-5p	1.8916	3.88E-06	3.00E-04	hsa-miR-424-3p	-6.3165	3.78E-11	3.27E-08
hsa-miR-141-3p	3.2814	6.62E-06	4.47E-04	hsa-miR-381-3p	-3.2038	6.92E-11	4.42E-08
hsa-miR-2861	1.4815	3.37E-05	1.55E-03	hsa-miR-4269	-4.1992	4.75E-10	2.24E-07
hsa-miR-6727-5p	1.3651	6.38E-05	2.61E-03	hsa-miR-377-5p	-3.2665	4.92E-10	2.24E-07
hsa-miR-6068	1.9206	7.03E-05	2.85E-03	hsa-miR-127-5p	-2.8872	1.05E-09	4.58E-07
hsa-miR-4532	1.7001	7.78E-05	2.98E-03	hsa-miR-127-3p	-2.4917	1.36E-09	5.11E-07
hsa-miR-1268a	1.5445	1.07E-04	3.95E-03	hsa-miR-487a-5p	-3.6043	2.00E-09	7.27E-07
hsa-miR-1268b	1.4048	1.46E-04	5.06E-03	hsa-miR-409-5p	-3.9076	2.64E-09	8.40E-07
hsa-miR-6824-5p	2.5470	1.80E-04	6.02E-03	hsa-miR-214-3p	-1.6237	3.65E-09	1.05E-06
hsa-miR-1227-5p	1.7438	3.25E-04	9.26E-03	hsa-miR-654-3p	-3.5810	7.41E-09	1.74E-06
hsa-miR-3141	1.6369	3.40E-04	9.58E-03	hsa-miR-412-5p	-3.0748	8.64E-09	2.00E-06
hsa-miR-3656	1.4694	4.24E-04	1.13E-02	hsa-miR-1185–2-3p	-3.4056	9.86E-09	2.25E-06
hsa-miR-6732-5p	1.5848	4.79E-04	1.25E-02	hsa-miR-1185–1-3p	-3.9161	1.23E-08	2.77E-06
hsa-miR-4467	1.8006	5.19E-04	1.32E-02	hsa-miR-21-5p	-3.5938	2.82E-08	5.45E-06
hsa-miR-8072	1.5614	5.22E-04	1.33E-02	hsa-miR-433-3p	-3.8969	3.49E-08	6.40E-06
hsa-miR-6756-5p	1.4758	5.33E-04	1.35E-02	hsa-miR-485-5p	-4.1670	6.40E-08	1.08E-05
hsa-miR-7108-5p	1.5724	6.44E-04	1.54E-02	hsa-miR-431-5p	-3.4643	1.29E-07	2.06E-05
hsa-miR-6805-5p	1.6830	7.26E-04	1.70E-02	hsa-miR-155-5p	-3.2221	1.48E-07	2.15E-05
hsa-miR-4728-5p	1.8029	7.44E-04	1.73E-02	hsa-miR-34a-5p	-1.8853	5.12E-07	5.83E-05
hsa-miR-6771-5p	1.4431	7.94E-04	1.80E-02	hsa-miR-29b-1-5p	-2.4037	7.56E-07	8.36E-05
hsa-miR-149-5p	2.5388	8.38E-04	1.82E-02	hsa-miR-31-5p	-6.5330	8.72E-07	9.28E-05
hsa-miR-6880-5p	1.6143	9.42E-04	1.99E-02	hsa-miR-485-3p	-3.5422	1.01E-06	1.01E-04

### 3.3 The DEMs screening results in Burns

**Fig 3:**
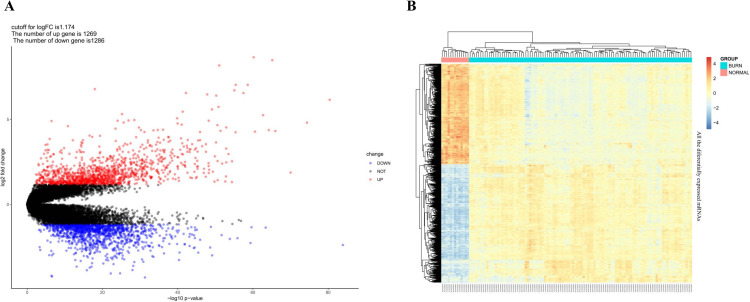
Volcano Plot and Heatmap of DEMs. A. Volcano Plot of DEMs. Red dots represent up-regulated genes, and blue dots represent down-regulated genes; B. Heatmap of DEMs. The left-most thirteen samples were sourced from the healthy control patients’ group, and the right-most one hundred and four samples were from the severe burn patients’ group. Blue color implies low expression, while red color implies high expression.

The expression levels of mRNAs in 104 severe burn patients and 13 healthy control individuals were investigated. The cutoff for log_2_FC of mRNAs is 1.174, 1269 (49.67%) up-regulated mRNAs and 1286 (50.33%) down-regulated ones identified. In Table 3, the top 25 up-regulated and top 25 down-regulated DEMs are outlined with corresponding log_2_FC, *P*-value, and FDR values. S3 Table with a complete file of DEMs was also provided. In Fig 3A, a volcano map illustrating the traits of all the DEMs on the correlation of –log_10_ (p-value) and log_2_FC was provided, together with a heat map of DECs as shown in Fig 3B from which the difference between healthy and severe burn patients’ groups was visually displayed. There is no significant difference in the number of changes between up-regulated and down-regulated mRNAs. However, the log_2_FC values of up-regulated mRNAs are significantly larger than those of down-regulated mRNAs, as shown in Table 3 and Fig 3A.

**Table 3:** The top 50 mRNAs in burn samples.

Top 25 up-regulated mRNAs				Top 25 down-regulated mRNAs			
Name	Log_2_FC	*P* Value	FDR	Name	Log_2_FC	*P* Value	FDR
MCEMP1	6.1515	3.91E-81	1.07E-76	NOV	-2.3850	1.26E-84	6.88E-80
S100A12	4.8098	4.20E-75	7.66E-71	GRAMD1C	-1.3584	4.02E-64	2.74E-60
S100A9	1.8728	9.77E-71	1.34E-66	EPHB1	-1.3414	2.37E-60	1.08E-56
GYG1	4.3156	1.13E-66	1.24E-62	TIGD3	-2.2649	4.43E-60	1.86E-56
CD177	8.4794	7.16E-66	6.52E-62	BTBD11	-1.9823	1.30E-56	3.96E-53
GYG1	4.3726	6.17E-65	4.82E-61	TRANK1	-2.9925	7.07E-50	8.41E-47
ANXA3	5.2463	2.03E-63	1.23E-59	MAP3K14	-1.3190	1.49E-49	1.70E-46
CYSTM1	4.2805	2.93E-61	1.60E-57	HSH2D	-2.5316	2.29E-49	2.55E-46
MMP8	8.6503	6.19E-61	3.07E-57	PDE4B	-2.8312	3.40E-46	2.78E-43
HP	7.0302	2.42E-59	9.46E-56	SPOCK2	-1.4840	7.77E-46	5.66E-43
GPR84	6.5471	3.26E-58	1.19E-54	EPHA4	-1.3546	2.51E-45	1.68E-42
MMP9	4.2406	4.07E-56	1.11E-52	LINC00877	-1.9767	2.62E-45	1.73E-42
ARG1	6.7087	8.56E-56	2.23E-52	CHST7	-1.8138	4.53E-45	2.81E-42
RAB32	2.5382	3.55E-55	8.44E-52	PTCH1	-2.5688	5.10E-45	3.13E-42
S100A8	1.3138	3.76E-55	8.56E-52	NOG	-1.7689	1.53E-44	9.18E-42
UPP1	2.9615	3.29E-54	6.91E-51	PTPN4	-1.3353	2.32E-44	1.37E-41
FCER1G	2.4722	1.24E-53	2.35E-50	NMT2	-1.7393	5.79E-44	3.30E-41
GADD45A	4.5281	1.24E-53	2.35E-50	PASK	-2.4429	6.73E-44	3.75E-41
NLRC4	3.2848	1.25E-53	2.35E-50	TMCC1	-1.9003	7.87E-44	4.30E-41
GAPDH	1.3502	2.23E-53	3.94E-50	SIN3A	-1.4092	1.97E-43	1.06E-40
METTL9	2.4041	1.51E-52	2.50E-49	MAN1C1	-2.3348	1.28E-42	6.20E-40
BMX	4.3281	4.19E-52	6.54E-49	ALDH1A1	-2.3448	1.45E-42	6.86E-40
MMP8	8.1695	8.86E-52	1.35E-48	TAGAP	-1.7319	2.64E-42	1.22E-39
CLEC4D	5.7315	1.44E-51	2.13E-48	CLIC3	-2.8260	3.22E-41	1.35E-38
GAPDH	1.4291	2.35E-51	3.38E-48	AUTS2	-3.2661	4.46E-41	1.84E-38

### 3.4 Construction of ceRNA regulatory network in Burns and Keloids

**Fig 4:**
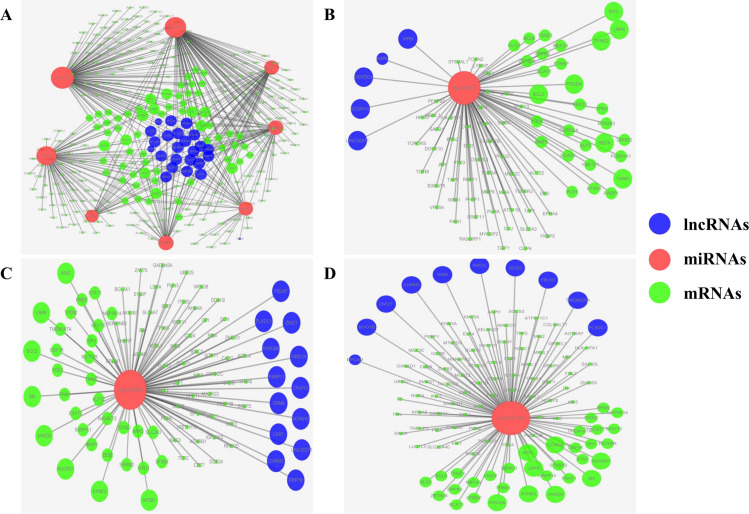
LncRNA-miRNA-mRNA ceRNA regulatory network in burns and keloids. A. Overall ceRNA Regulatory Network; B. The Sub-network of hsa-miR-21-Mediated ceRNA Regulatory Network; C. The Sub-network of hsa-miR-34a-Mediated ceRNA Regulatory Network; D. The Sub-network of hsa-miR-155-Mediated ceRNA Regulatory Network. DELs, DEMis, and DEMs are represented by blue, red, and green circles, respectively.

To further clarify the interactions among DELs, DEMis, and DEMs, the ceRNA regulatory network of burns was constructed, which facilitated a better understanding of the pathological processes of burns and keloids. Initially, LncBase Predicted v.2 was exploited to forecast the interaction between lncRNAs and miRNAs, which was further validated by the RNAhybrid program. Among the top 50 DEMis, 8 miRNAs were found to interact with 23 DELs, as identified by the limma package. Subsequently, the target mRNAs of 8 DEMis were retrieved from MiRBase, MirTarBase, and Targetscan. 330 DEMs were obtained for interacting with 8 miRNAs above. Subsequently, a ceRNA regulatory network of burns was reconstructed by incorporating 23 DELs, 8 DEMis and 330 DEMs, as shown in Fig 4A. Further, the sub-networks were analyzed based on the gene degrees within the ceRNA network. Hsa-miR-21, hsa-miR-34a, and hsa-miR-155 were identified as the most significant miRNAs associated with the pathological progress of burns and keloids. Sub-networks for these three miRNAs were constructed, as shown separately in Figs 4B, 4C, and 4D. A total of 87 nodes could be identified as hub nodes in hsa-miR-21associated ceRNA network, including 5 lncRNAs and 81 mRNAs, see from Fig 4B. 13 lncRNAs and 90 mRNAs were in hsa-miR-34a associated ceRNA network, as shown in Fig 4C. 10 lncRNAs and 107 mRNAs participated in building hsa-miR-155 associated ceRNA network, as shown in Fig 4D.

### 3.5 . Functional enrichment analysis of DEMs in sub-networks

**Fig 5:**
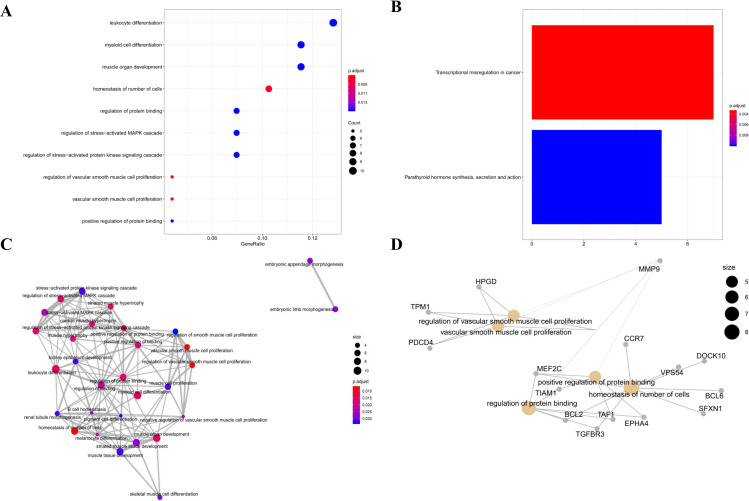
Functional Enrichment Analysis of the hsa-miR-21-Mediated Sub-ceRNA Network. A. The Top 10 Enriched GO BP Terms; B. The Enriched KEGG Pathways; C. The GO Interaction Network; D. The cnetplot of GO Terms.

The ClusterProfiler package was utilized to perform KEGG and GO Biological Process (BP) analysis of DEMs in the sub-networks, providing a clear understanding of the mechanisms underlying burns and keloids. In the hsa-miR-21-mediated sub-network, the top 10 GO BP terms with the most significant *P*-values are presented in Fig 5A. Notably, leukocyte differentiation, myeloid cell differentiation, muscle organ development, cellular homeostasis, regulation of protein binding, regulation of stress-activated MAPK cascade, regulation of stress-activated protein kinase signaling cascade, vascular smooth muscle cell proliferation, and positive regulation of protein binding were identified as key factors in the pathological evolution of burns. Transcriptional misregulation in cancer and parathyroid hormone synthesis, secretion and action were targeted as the most important pathways regulating the process of burns, as shown in Fig 5B. Additionally, we constructed the GO interaction network and the cnetplot of GO terms, both of which are displayed in Figs 5C and 5D.

**Fig 6:**
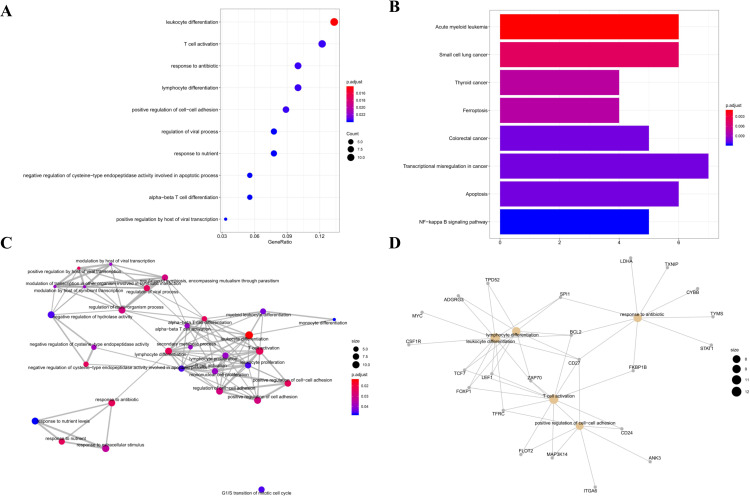
Functional Enrichment Analysis of the hsa-miR-34a-Mediated Sub-ceRNA Network. A. The Top 10 G) B) Terms; B. The Enriched KEGG Pathways; C. The GO Interaction Network; D. The cnetplot of GO Terms.

In the hsa-miR-34a-mediated sub-network, the top 10 GO BP terms, encompassing leukocyte differentiation, T-cell activation, response to antibiotics, lymphocyte differentiation, positive regulation of cell-cell adhesion, regulation of viral processes, response to nutrients, negative regulation of cysteine-type endopeptidase activity involved in apoptotic processes, alpha-beta T-cell differentiation, and positive regulation of viral transcription by the host, are presented in Fig 6A. Acute myeloid leukemia, small cell lung cancer, thyroid cancer, ferroptosis, colorectal cancer, transcriptional misregulation in cancer, apoptosis and the NF-kappa B signaling pathway were identified as the most important 8 pathways regulating the process of burns, as shown in Fig 6B. GO interaction network and the cnetplot of GO terms were also exhibited in Figs 6C and 6D.

**Fig 7:**
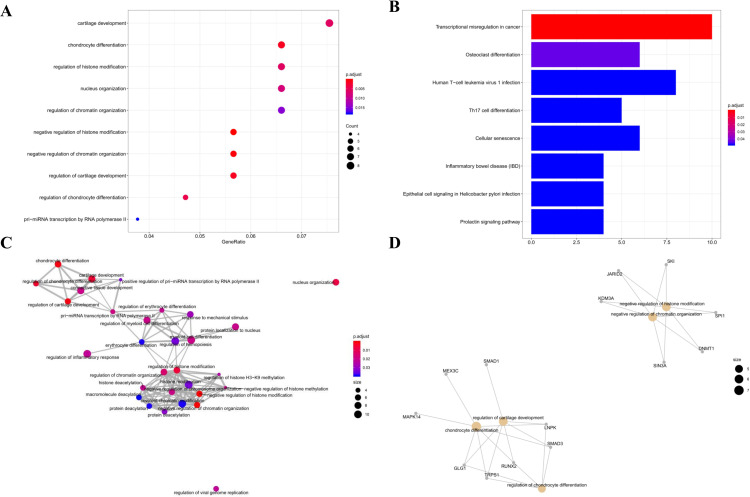
Functional Enrichment Analysis of the hsa-miR-155-Mediated Sub-ceRNA Network. A. The Top 10 Enriched GO BP Terms; B. The Enriched KEGG Pathways; C. The GO Interaction Network; D. The cnetplot of GO Terms.

The first 10 GO BP terms in the hsa-miR-155 mediated sub-network are shown in Fig 7A. The pathological development of burns is associated with cartilage development, chondrocyte differentiation, regulation of histone modification, nuclear organization, regulation of chromatin organization, negative regulation of histone modification, negative regulation of chromatin organization, regulation of cartilage development, regulation of chondrocyte differentiation, and pri-miRNA transcription mediated by RNA polymerase II. Transcriptional mis-regulation in cancer, osteoclast differentiation, human T-cell leukemia virus infection, Th17 cell differentiation, cellular senescence, and inflammatory bowel disease (IBD) *etc.* were identified as the most important pathways in burns, as shown in Fig 7B. The GO interaction network and the cnetplot of GO terms were also constructed, which are displayed in Figs 7C and 7D.

## 4. Discussion

An estimated 180,000 deaths occur annually as a result of burn injuries, according to the WHO ^3^. Considering the high mortality rate associated with burn injuries, burns are of sufficient severity to attract the attention of the entire society [30]. The burned skin is often accompanied by excessive proliferation of fibrotic tissue, which leads to the formation of HS/keloid. These occur in approximately 70% - 80% of post-burn cases [31]. Due to pain, itch, and their aesthetic burden, HS and Keloids are putting a great threat to patients with burns [32].

Initially, the ceRNA network of burns was constructed based on transcriptomics data. Following a systematic analysis of the datasets obtained from the GEO database, three long lncRNAs, namely lnc-WRB, lnc-SCNN1G, and LINC00271, and three miRNAs, namely hsa-miR-21, hsa-miR-34a, and hsa-miR-155, were identified as key factors in regulating the burn process. lnc-WRB, lnc-SCNN1G, and LINC00271 are three long non-coding RNAs that may play important regulatory roles in the burn process. Specifically. lnc-WRB may be associated with key processes such as inflammation, wound healing, and tissue repair in the burn process. It may affect the biological response after burns by regulating the expression of specific genes or participating in cellular signaling pathways. lnc-SCNN1G may be associated with physiological regulation such as water and salt balance and intracellular sodium channel function after burns. It may play a role in ion exchange and homeostasis regulation within cells after burns. LINC00271 may be involved in the regulation of processes such as cell apoptosis, proliferation, and repair after burns. It may affect the regeneration and repair of burn tissues by regulating the cell cycle, gene transcription, or other pathways. The specific relationship between these lncRNAs and burns needs to be further confirmation and elucidation through additional experimental research and exploration of molecular mechanisms. By exploring their functions and mechanisms in the context of burns, a deeper understanding and potential targeted therapeutic strategies for burn treatment and management can be provided. Hsa-miR-21 may be involved in regulating the inflammatory response, tissue repair, and cell proliferation after burns. It may influence the biological response of burn tissues, facilitating burn healing and repair. Hsa-miR-34a is generally regarded as a tumor-suppressing miRNA, which may play a regulatory role in cell apoptosis and tissue repair during burn healing. Hsa-miR-155 may participate in regulating anti-inflammatory responses and immune reactions after burns, helping to control the degree of inflammation and tissue repair processes. The specific relationship between these miRNAs and burns needs to be further confirmed and elaborated through additional experimental studies. Nevertheless, they may offer potential targeted treatment strategies for burn treatment and management, contributing to a deeper understanding of the molecular mechanisms of burn healing. Guo et al. suggested a rosy prospect of the therapeutic scheme targeting miRNAs for treating fibrotic diseases, based on the notable therapeutic effect of miR‑21 on HS [33]. In addition, Zhou et al. reported that miR-21/Smad7 talking promoted collagen production in keloid [34].

Within the ceRNA regulatory network, 330 mRNAs, mainly encompassing TGF-β family, Smad family, MMP family, IGF family, CD family, and IL-family, were filtered as candidate genes associated with the pathological progression of burns. TGF-β1 is beneficial for collagen deposition, which promotes fibroblast proliferation [35], and the TGF-β1/Smads pathway facilitates fibrosis [36]. Chen et al. discovered that TLR4 in keloids upregulated the expression of TGF-β through the Smad4 signaling pathway [37]. Phan et al. clearly demonstrated that epidermal-dermal interactions in keloids result in activation of the TGFβ-Smad axis of which Smad signaling, especially Smad3 plays a crucial pro-fibrotic role in keloids pathogenesis that may develop as an important drug target for treating keloid [38]. After, Chen et al. proposed that TGF-β1 was a key factor in inhibiting cell proliferation and collagen synthesis in keloids [39]. Xin and his team reported that the expansion of CD26^ +^ fibroblasts was responsible for keloid progression [40]. Zhu et al. indicated that a signaling axis consisting of lncRNA-ATB/miR200c/ZNF217/TGF-β2 governs the autocrine secretion of TGF-β2 in keloids [41].

Surprisingly yet interestingly, genes within the networks were screened, and several cancer-related pathways were identified, including small-cell lung cancer, transcriptional misregulation in cancer, thyroid cancer, and colorectal cancer. These were recognized as important pathways regulating the burn process. Considering these questions, relevant experimental and/or clinical validation was sought. It was found that tamoxifen, which has been widely used in breast cancer treatment over the past 20 years, represents a potential option for promoting wound healing [42, 43]. Altering RNA transcription, decreasing cellular proliferation, delaying or arresting cells in the G1 phase, and interfering with multiple genes such as TGF-β and insulin growth factor (IGF) were the relevant mechanisms of tamoxifen for treating keloids [43]. Fluorouracil (5-FU), a chemotherapeutic drug that blocks DNA replication, is another anti-cancer drug that is recommended for the treatment of keloids. In the review by Bijlard et al., the use of 5-FU in the treatment of keloid in 482 patients achieved a good or excellent outcome in 45%-78% [32]. The common underlying mechanism between keloids and cancer is the malignant growth pattern of cells. This has led clinical doctors to use chemotherapeutic agents for keloid treatment, which is partly why numerous tumor-related pathways are present in the above-constructed network [44].

In addition to their potential involvement in the pathological progression of burns, hsa-miR-34a and hsa-miR-155 are also predominantly reported to be involved in tumors. Bonetti et al. revealed that the miR-34 family, and miR-34a particularly, serves to inhibit the expansion of the pool of mammary stem cells and early progenitor cells, in a p53-independent manner. The activation of miR-34a-dependent programs may offer a therapeutic opportunity for the subset of breast cancers that exhibit poor responses to conventional therapies [45]. Lan et al. found that promoter methylation of miR-34a and miR-34b/c occurs frequently in breast and lung cancers and put forward that methylation of miR34a may serve as a marker for breast cancer [46, 47]. Hsa-miR-34a is also reported to be involved in ovarian cancer [48], breast cancer [49], the DNA damage response [50], liver fibrosis [51], cancer metastasis circuits [52], colon carcinogenesis [53], hepatocarcinogenesis [54] and pancreatic cancer [55] *etc*. Deficiency of host miR-155 promoted overall antitumor immunity by the accumulation of functional myeloid-derived suppressive cells in the tumor microenvironment [56]. Wu et al. said that the miR-155HG/miR-155 axis is very important in facilitating glioma progression, which can be considered as a prognostic factor for patient survival in glioblastoma [57]. Costinean et al. showed that the incidence of frank B cell malignancies is high in E-mmu-miR-155 transgenic mice, indicating that miR-155 plays an important regulatory role in tumorigenesis [58]. Keloids and cancer share certain common mechanisms during their pathological processes. Although experimental evidence is lacking, it is reasonable to postulate that many anti-tumor drugs are also effective in treating burns, as well as HS and keloids post-burns.

In summary, miRNA targeted therapy uses synthetic miRNA molecules to regulate abnormal gene expression and achieve disease treatment. The treatment process encompasses identifying target miRNAs and target genes, designing and synthesizing miRNA molecules, delivering miRNAs to target cells, targeting and regulating gene expression, and evaluating treatment effects. miRNA-targeted therapy presents several advantages, such as high specificity, reversibility, and the capacity for multi-target regulation. Nevertheless, it still has to confront challenges like miRNA delivery, safety issues, and the validation of clinical applications.

## 5. Conclusions

Collectively, all nodes within the sub-ceRNA network exert either a direct or an indirect influence on the pathological processes associated with burns and keloids. This study provides a novel perspective on the DELs-DEMis-DEMs ceRNA network, deepening our understanding of the ceRNA regulatory mechanisms underlying the pathogenesis of burns and keloids. Nevertheless, systematic and rigorous experimental validations are indispensable for validating our findings.

## Supporting information

S1 Data Source code. Supplementary files-Burn-ceRNA.
(RMD)

S1 Table Differentially expressed lncRNA. (XLSX)

S2 Table Differentially expressed miRNA. (XLSX)

S3 Table Differentially expressed mRNA. (XLSX)
